# Von Willebrand disease type Vicenza: In search of a classification for the archetype of reduced von Willebrand factor survival

**DOI:** 10.1002/jha2.196

**Published:** 2021-05-05

**Authors:** Alessandra Casonato, Eva Galletta, Federico Galvanin, Viviana Daidone

**Affiliations:** ^1^ Department of Medicine University of Padua Medical School First Chair of Internal Medicine Padua Italy

**Keywords:** type 1 von Willebrand disease, type Vicenza VWD, von Willebrand factor, VWD classification, VWF survival

## Abstract

Type Vicenza von Willebrand disease (VWD) features a von Willebrand factor (VWF) with a very short half‐life, and is classified as a form of type 1 VWD. To test the appropriateness of type Vicenza VWD classification, the main features of 17 patients from eight unrelated families were analysed. They had low VWF antigen levels and function (always below 20 U/dl); ristocetin‐induced platelet aggregation sometimes normal, sometimes reduced/absent (even in the same patient); normal platelet VWF levels; an increased VWF propeptide to VWF antigen ratio (8.74 ± 1.65 vs. normal 1.04 ± 0.28) and a reduced VWF half‐life. Plasma VWF multimer levels were homogeneously reduced, and unusually large VWF multimers were sometimes present. Recombinant p.R1205H VWF showed a normal synthesis, release, function, and multimer pattern, with no ultra‐large VWF multimers. The mathematical model by Galvanin et al. was used to explore the kinetic changes in VWF after DDAVP. It showed that the release, but especially the proteolysis (*k*_proteol_ 1.0^−3^ ± 2.5^−3^ vs. normal 4.5^−4^ ± 6.4^−4^) and elimination (*k*_el_ 1.0^−2^ ± 5.2^−3^ vs. normal 1.1^−3^ ± 6.8^−4^) of type Vicenza VWF were significantly higher than normal. The increased elimination is consistent with the short half‐life, while the increased proteolysis was unexpected. As a shorter survival of VWF is wholly responsible for the type Vicenza VWD phenotype (VWF synthesis, structure and function are normal), it might be better to classify it as a type 2 VWD (rather than type 1) to emphasise the greater interaction with clearance receptors as a new VWF functional defect.

## 1 INTRODUCTION

Platelet plug formation at the site of vascular injury is guaranteed by the contribution of von Willebrand factor (VWF), a multimeric glycoprotein capable of forming a bridge between the subendothelial matrix and platelets, as well as promoting platelet interactions [1]. The main feature of VWF is its multimeric structure, with oligomers ranging from 500 kDa to more than 20 million Daltons, with the largest forms having the highest haemostatic capacity [2]. A defective VWF identifies von Willebrand disease (VWD), the most common inherited bleeding disorder. VWD can be characterised by quantitative VWF defects (type 1 and type 3) or VWF functional abnormalities (type 2) [3].

Type 1 VWD is defined as a partial quantitative VWF defect, with the residual VWF featuring no functional abnormalities, and a normal or near‐normal multimer pattern [4, 5, 6].

When type Vicenza VWD was first described by Mannucci et al. in 1985 [7], it was defined as a ‘platelet‐normal type 1 VWD’, underscoring that the normal platelet VWF content coincided with a significant reduction in circulating VWF. The large VWF multimers were all present, sometimes with the presence of unusually large VWF oligomers as well [8]. It was later reclassified as type 2M VWD because of the abnormal ristocetin‐induced platelet aggregation (RIPA) seen in these patients. The defect was found associated with the p.R1205H mutation [9, 10], usually combined with the p.M740I mutation [11], which is now considered a polymorphism. It was ultimately demonstrated that type Vicenza VWD is characterised by a very short VWF survival—the main reason for the low circulating VWF levels [12]. An accelerated VWF clearance seems to be the only explanation for the presence of ultra‐large multimers in type Vicenza VWD [13]. It is now classified once again as a type 1 VWD, based on low circulating VWF levels and the presence of all VWF multimers [3].

Ever since type Vicenza VWD was first described, its classification has been evolving, and a final solution probably has yet to be found [14]. In the present contribution, we analyse all the known features of VWF in cases of type Vicenza VWD to establish the appropriateness of its current classification.

## 2 MATERIALS AND METHODS

Patients and normal subjects were studied in accordance with the Helsinki Declaration, after obtaining their written informed consent.

### 2.1 Haemostatic analysis

The main haemostatic findings in the patients considered here have been reported elsewhere [15]. Plasma and platelet VWF antigen (VWF:Ag) was measured by enzyme‐linked immunosorbent assay (ELISA) using horseradish peroxidase (HRP)‐conjugated anti‐VWF polyclonal antibody (Dako, Glostrup, Denmark) [16]. VWF collagen‐binding (VWF:CB) activity was assessed with an ELISA test using type III collagen (Sigma, Milan, Italy). Factor VIII coagulant (FVIII:C) was measured using a single‐stage method with cephaloplastin as activated cephalin. DDAVP (1‐desamino‐8‐D‐arginine vasopressin; Emosint, Kedrion, Italy) was administered subcutaneously at a dose of 0.3 μg/kg. Blood samples were collected before and 15, 30, 60, 120, 180, 240, 360 and 480 min, and 24 h after administering DDAVP. Time courses of factor VWF:Ag and VWF:CB plasma concentrations after DDAVP administration were analysed according to a one‐compartment model with first‐order input and output kinetics [17]. VWF propeptide (VWFpp) was measured using a home‐made ELISA method. Briefly, diluted reference and patient plasma samples were added to microwells on microtitration plates coated with a monoclonal antibody specific for VWFpp (CLB‐Pro 35, Sanguin, Netherlands). After 2 h of incubation at 37°C, the bound VWFpp was assessed with a second anti‐VWFpp HRP‐labelled monoclonal antibody (M193904, Sanguin). The results are given in U/dl, taking the first reference curve dilution as 100 U/dl [18]. VWF multimers were analysed by electrophoresis on 1.6% high‐gelling temperature agarose containing 0.1% sodium dodecyl sulphate. The multimers were detected by autoradiography after reaction with anti‐VWF polyclonal antibody (DAKO) labelled with ^125^I, and viewed with the DS‐50000 Epson densitometer scanner.

### 2.2 Genetic analysis

Genomic DNA was extracted from peripheral blood leukocytes using the QIAamp DNA Blood Mini Kit (Qiagen, Hilden, Germany). The *VWF* gene exons were amplified and sequenced using primers chosen according to the NM_000552 *VWF* sequence. PCR amplification and sequencing of the VWF gene was performed, as previously described [19].

### 2.3 Expression experiments

The pSVvWFA plasmid containing normal human full‐length *VWF* cDNA was mutated by recombinant PCR, as previously described [20]. For the expression studies, the pSVH1205 VWF was transiently transfected into baby hamster kidney (BHK) cells stably transfected with furin (FUR4BHK) using the Fugene transfection reagent (Roche, Mannheim, Germany). Co‐transfections with pSVvWFA and pSVH1205 VWF constructs were used to mimic the patient's heterozygous state. After 72 h, the transfection media containing VWF were removed, and the VWF:Ag and VWF:RCo values were quantified. The results of each transfection were calculated as the mean of six replicates. The results obtained were expressed as a percentage, taking 100 as the wild‐type VWF values.

### 2.4 Mathematical model

The time course of post‐DDAVP VWF:Ag and VWF:CB was analysed with a two‐compartment, physiologically based model derived from the one proposed by Galvanin et al. [21], which can characterise the release, proteolysis and clearance mechanisms of VWF, and its multimer distribution (see Supporting Materials). This model comprises a system of differential and algebraic equations, and each subject is characterised by three main pharmacokinetic (PK) constants: the VWF release rate constant *k*_rel_, the proteolysis rate constant *k*_proteol_, and the elimination rate constant *k*_el_—the latter is assumed to be the same for ultra‐large plus high‐molecular weight (UL+HMW) multimers as for low‐molecular weight (LMW) multimers. The model is based on the assumptions that (a) HMW and LMW multimers are present in the basal state and/or after DDAVP; (b) UL and HMW multimers can be cleaved to form LMW multimers; (c) we can judge the quantities of UL+HMW+LMW multimers from VWF:Ag measurements; and (d) VWF:CB gives us a measure of the quantity of UL+HMW multimers.

### 2.5 Statistical analysis

Laboratory data were expressed as mean ± standard deviation (SD). The Student's *t*‐test was used to compare all results, and Pearson's correlation analysis was conducted to assess the association between the RIPA and VWF:Ag parameters. The *p*‐values below .05 were considered statistically significant.

## 3 RESULTS

### 3.1 Patients

Seventeen patients with type Vicenza VWD were studied. They belonged to eight unrelated families and all but one came from north‐east Italy. Fourteen patients were carrying both the p.R1205H and the p.M740I mutations, while three patients only had the p.R1205H mutation, all at heterozygous level. Patients were in non‐O blood groups, except for those carrying the p.R1205H mutation alone. Bleeding symptoms were quantified by the ISTH bleeding assessment tool (BAT): the scores were higher in patients than in healthy controls, with no difference between females (10.5 ± 5.1 vs. normal range 0–5) and males (11.5 ± 6.0 vs. normal 0–3). Table 1 shows the patients’ main haemostatic findings.

**TABLE 1:** Main haemostatic findings in the sample of patients with type Vicenza VWD

**Patients**	**Family**	**Age/blood group**	**RIPA 1.2 mg/ml%**	**VWF:Ag U/dl**	**VWF:CB U/dl**	**VWF:CB ratio**	**VWF:RCo U/dl**	**VWF:RCo ratio**	**FVIII:C U/dl**	**FVIII:C ratio**	**VWF:pp U/dl**	**VWFpp ratio**	**Plat. VWF:Ag U/dl**	**Mutations**
1	I	50/O	38.1 (60.6‐15)	9.2	9.8	1.10	11.5	1.25	12.5	1.35	53.5	8.23	86.6	p.R1205H
2	II	71/O	5.1 (18.6)	5.5	4.8	0.87	5.0	0.90	12.0	2.1	NP	NP	125	p.R1205H
3	III	43/O	NP	11.2	9.7	0.87	11.5	1.02	12.2	1.1	81.4	7.30	NP	p.R1205H
4	IV	60/AB	71.0	13.8	11.4	0.83	15.1	1.10	20.0	1.44	125.6	9.10	95.8	p.R1205H+p.M740I
5	IV	36/B	51.0	18.4	19.6	1.10	19.7	1.07	34.2	1.85	130.9	7.10	77.0	p.R1205H+p.M740I
6	V	52/A	6.9 (73.4)	14.5	12.5	0.87	10.7	0.74	24.1	1.66	130.5	9.0	67.0	p.R1205H+p.M740I
7	VI	35/AB	0 (72.5)	8.7	7.6	0.88	8.5	0.98	23.0	2.6	47.6	6.80	76.2	p.R1205H+p.M740I
8	VI	22/AB	26.6 (66.4)	8.2	7.0	0.85	11.2	1.36	17.1	2.1	61.6	8.67	85.6	p.R1205H+p.M740I
9	VI	0.1/B	NP	3.8	3.7	1.0	NP	NP	12.5	3.2	NP	NP	NP	p.R1205H+p.M740I
10	VII	57/B	0	13.8	12.2	0.88	11.8	0.85	17.5	1.26	134.8	9.76	73.0	p.R1205H+p.M740I
11	VII	30/A	NP	9.75	8.3	0.85	5.1	0.52	22.9	2.34	130.6	13.40	95.0	p.R1205H+p.M740I
12	VII	52/A	39.0	7.2	6.9	0.95	6.0	0.83	15.5	2.15	60.6	8.42	77.3	p.R1205H+p.M740I
13	VII	33/A	8.4	9.9	13.8	0.93	7.4	0.74	17.0	1.71	103.1	10.42	86.4	p.R1205H+p.M740I
14	VII	65/A	8.1 (70)	14.8	13.8	0.93	13.5	0.91	19.6	1.32	146.8	9.90	91.5	p.R1205H+p.M740I
15	VII	32/A	64.6	10.3	8.1	0.78	6.8	0.85	22.1	2.14	60.8	8.34	95.1	p.R1205H+p.M740I
16	VIII	68/A	17.7 (63)	18.2	18.4	1.00	15.4	0.85	38.0	2.1	163.8	9.10	89.8	p.R1205H+p.M740I
17	VIII	56/A	78.7	15.3	NP	NP	16.0	1.04	30.5	1.99	110.6	7.40	78.5	p.R1205H+p.M740I
Normal values			60–84	60–160	65–150	>0.75	60–130	>0.75	60–160	>0.75	70.9–152.9	0.76–1.38	70–140	

*Note*: RIPA: additional test results are included in brackets. VW:CB ratio = VWF:CB/VWF:Ag. VWF:RCo ratio = VWF:RCo/VWF:Ag. FVIII:C ratio = FVIII:C/VWF:Ag. VWFpp ratio = VWFpp/VWF:Ag. All patients are heterozygous for the p.R1205H and p.M740I mutations.

Abbreviation: NP = not performed.

### 3.2 VWF synthesis is normal in type Vicenza VWD

As shown in Table 1, plasma VWF:Ag was significantly reduced, with values ranging between 3.8 U/dl and 18.4 U/dl. Patients’ platelet VWF content was always within normal range, indicating a normal VWF synthesis (Table 1). This latter finding was confirmed by in vitro expression of the VWF molecule, which revealed recombinant 1205H‐VWF antigen values indistinguishable from those of wild‐type VWF, whether the mutation was expressed at homozygous (97.6%) or heterozygous (109.3%) level.

### 3.3 VWF function is normal in type Vicenza VWD

RIPA useful for exploring the interaction between VWF and platelet GPIb, was found sometimes normal, sometimes reduced, even in the same patient (Table 1). There was no statistical correlation between RIPA values and VWF:Ag (*p* = .467), suggesting that platelet responsiveness to ristocetin is uninfluenced by patients’ higher or lower VWF:Ag levels. VWF:CB (exploring VWF binding to collagen and correct multimer structure), and VWF:RCo (exploring the VWF interaction with platelet GPIb and multimer structure) were both reduced, but normal when expressed as ratios of VWF:CB/VWF:Ag and VWF:RCo/VWF:Ag, confirming that there are no VWF functional abnormalities in type Vicenza VWD. The same behaviour was seen in the recombinant VWF, which showed normal VWF:RCo, whether expressed as an absolute value or ratio at heterozygous (96.3%) or homozygous (94.7%) level.

### 3.4 VWF multimer structure is normal in type Vicenza VWD

**FIGURE 1:**
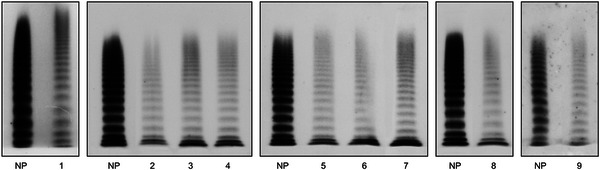
Plasma VWF multimer pattern, obtained using 1.6% agarose gel in nine patients with type Vicenza VWD as compared to normal pooled plasma (NP). High‐molecular weight multimers are at the top and low‐molecular weight multimers at the bottom. Note the presence of ultra‐large VWF multimers in patients 1 and 9

**FIGURE 2:**
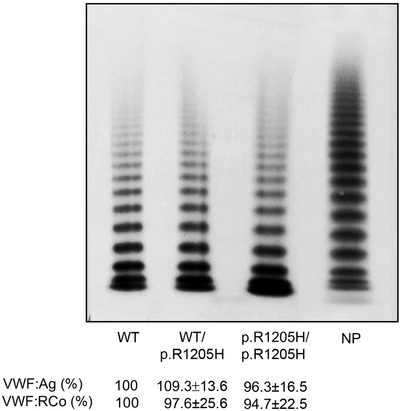
Multimer pattern of recombinant VWF expressing the p.R1205H type Vicenza mutation at heterozygous and homozygous level, compared to wild‐type (WT) VWF and normal pooled plasma (NP). No difference in VWF:Ag, VWF:RCo and multimers pattern and no ultra‐large VWF multimers are evident in recombinant p.R1205H VWF compared to wild‐type (WT) VWF

Plasma VWF multimer analysis, performed under low‐resolution conditions, showed a consistent reduction in VWF levels, with all oligomers present and, in some instances, unusually large VWF multimers as well. Figure 1 shows the pattern for nine patients, but it was much the same in the other patients studied. The multimer pattern of the recombinant 1205H‐VWF was also normal, whether the VWF defect was expressed at heterozygous or homozygous level (Figure 2), and no ultra‐large VWF multimers were ever detectable.

### 3.5 VWF survival is significantly decreased in type Vicenza VWD

VWF survival was explored using both VWFpp and the DDAVP test. Patients’ mean VWFpp levels were indistinguishable from the normal counterpart (102.8 ± 38.5 U/dl vs. normal 111.94 ± 41.1 U/dl), while the VWFpp/VWF:Ag ratios (VWFpp ratio) were significantly increased (mean 8.74 ± 1.65 vs. normal 1.04 ± 0.28) (*p* < .001). No differences emerged between patients carrying the p.R1205H mutation alone and those carrying the p.M740I mutation as well (8.71 ± 1.95 vs. 8.46 ± 1.4). These two groups also coincided with subjects in the O and non‐O blood groups, respectively.

There was also evidence of a short VWF:Ag half‐life (*T*_½_el 1.26 ± 0.41 h vs. normal 16.0 ± 14.9 h) and VWF:CB half‐life (1.27 ± 0.48 h vs. normal 11.9 ± 10.0 h) calculated according to a one‐compartment model. There was no difference in VWF:Ag half‐life in relation to ABO blood group, neither for VWF:Ag nor for VWF:CB (Table 2).

**TABLE 2:** Mean VWF *T*_1/2_ elimination rate explored with a one‐compartment model in patients with type Vicenza VWD compared with healthy controls

**Subjects**	***T*_1/2_ elimination**					
	**VWF:Ag (h)** Mean ± SD			**VWF:CB (h)** Mean ± SD		
	**O + non‐O**	**O**	**non‐O**	**O + non‐O**	**O**	**non‐O**
Vicenza patients^a^	1.26 ± 0.41	1.61 ± 0.60	1.23 ± 0.30	1.27 ± 0.48	1.63 ± 0.86	1.15 ± 0.31
Healthy controls	16.0 ± 14.9	10.1 ± 4.6	21.7 ± 9.7	11.9 ± 10.0	8.1 ± 3.6	16.50 ± 8.34

^a^Type Vicenza patients in the O blood group were carrying the p.R1205H mutation alone; those in the non‐O blood groups carried both the p.R1205H and p.M740I mutations.

### 3.6 Post‐DDAVP multimer behaviour differs from normal in type Vicenza VWD

**FIGURE 3:**
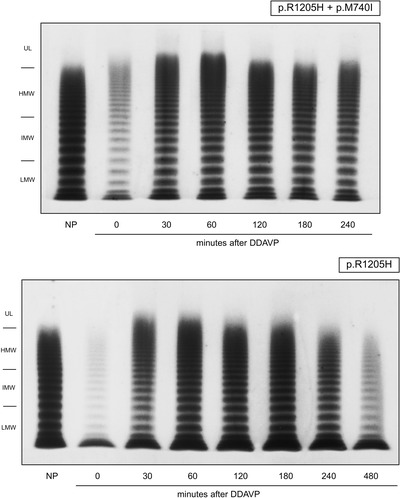
Post‐DDAVP multimer pattern in patients carrying both the p.R1205H and the p.M740I mutations (patient 6 in Figure 1) or the p.R1205H mutation alone (patient 2 in Figure 1). The pattern is also representative of those seen in the other patients studied

**FIGURE 4:**
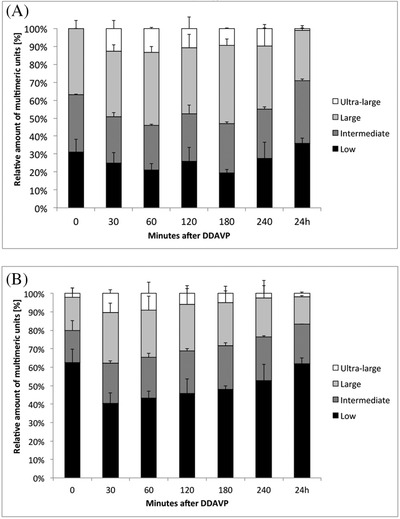
Relative amounts of multimers over post‐DDAVP time (HMW, high molecular weight; UL, ultra‐large; IMW, intermediate molecular weight; LMW, low molecular weight) after DDAVP challenge, as quantified with the ImageJ software from the photographic plate (stacked bars) in a type Vicenza patient (A) and a normal subject (B). The values are expressed in percentage, taking the total multimers quantity as 100%. Note the relative increase in the representation of LMW multimers in type Vicenza VWD before DDAVP infusion, their rapid decrease 30 min afterwards, and the inverse relationship between high‐ and low‐molecular weight VWF multimers. The latter findings confirm the increased proteolysis of type Vicenza VWF observed by the mathematical model after DDAVP, which does not occur in normal subjects

Post‐DDAVP multimer patterns showed an increase in the overall VWF levels, and the appearance of ultra‐large VWF multimers (Figure 3), that peaked at 30 min, then the large and ultra‐large multimers began to decrease from 60 min onwards. There were no differences in multimer patterns between patients with the p.R1205H mutation alone and those with the p.M740H as well (Figure 3). The relative proportions of LMW, intermediate‐molecular weight (IMW), HMW and UL multimers were quantified with a densitometric analysis using the ImageJ software, and expressed as mean ± standard deviation. The pertinent results are shown in Figure 4. Before DDAVP, the LMW VWF multimers were the most represented, and in higher than normal proportions (63% vs. normal 31%) compared to the intermediate and large forms. After DDAVP, the proportion of LMW forms became relatively lower at 30 min, then gradually increased again. In normal subjects on the other hand, the intermediate and large multimers remained prevalent, and the proportion of LMW oligomers decreased slightly after DDAVP (Figure 4).

### 3.7 Mathematical model demonstrates elimination, proteolysis and release of type Vicenza VWF is increased

**FIGURE 5:**
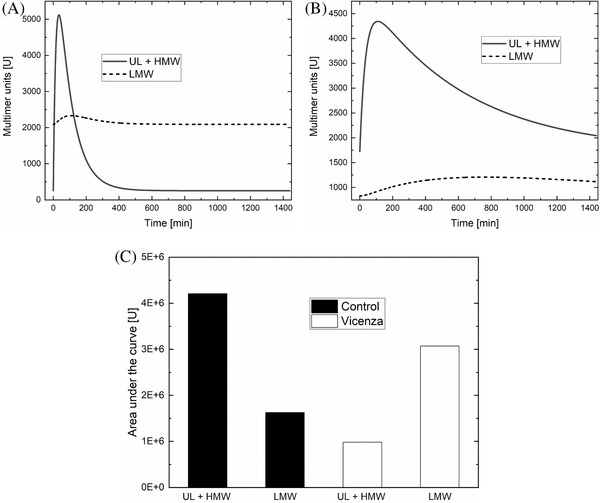
Time course of the amount of ultra‐large plus high‐molecular weight (UL+HMW) multimers (solid line) and low‐molecular weight (LMW) multimers (broken line) after DDAVP infusion as quantified by the model in patients with type Vicenza VWD (A), as compared with healthy controls (B). Area under the curve (AUC) for (UL+HMW) and LMW multimers after DDAVP infusion (C) as quantified by the model in patients with type Vicenza VWD (white bars) and healthy controls (black bars)

The metabolic pathways related to VWF after its release from endothelial cells—that is, release, proteolysis and elimination—were studied using the physiology‐based mathematical model developed by Galvanin et al. [18]. As shown in Table 3 and Figure 5, the model identified a substantial increase in patients’ VWF elimination rate constant (*k*_el_ parameter, 1.08^−2^/min vs. 1.17^−3^/min in normal subjects), VWF proteolysis rate constant (*k*_proteol_, 1.04^−3^/min vs. 4.59^−4^/min in normal) and VWF release rate constant (*k*_rel_, 5.87^−2^/min vs. normal 2.74^−2^/min). In other words, all the above pathways seemed to be accelerated, especially the elimination one. The model was also able to quantify the amounts of LMW and UL + HMW multimers before and after DDAVP administration using the VWF:Ag and VWF:CB values. Values of *T*_1/2_ estimated by the model are in good agreement with the observed data post‐DDAVP, while the total amount of VWF released (Q) is estimated to be, on average, higher in Vicenza subjects as compared to normal subjects. As seen in Figure 5, the post‐DDAVP behaviour of the VWF multimers was characterised by a sudden increase in large and ultra‐large multimers and a later less pronounced peak of the LMW forms, which occurred when the large forms were already decreasing. The model also showed that patients’ large and small multimers disappeared at the same rate (Figure 5A). In contrast, in normal subjects (Figure 5B) the post‐DDAVP increase of large multimers was greater than the one of small multimers, with both oligomers cleared more slowly. Analysing the area under the curve (AUC) of UL+HMW and LMW multimers (Figure 5C) suggests that in type Vicenza patients, the smaller multimers are derived by proteolysis from the larger ones, explaining why our patients’ LMW VWF multimers were relatively more represented than the larger forms, as well as their increased proteolysis rate constant.

**TABLE 3:** Post‐DDAVP average PK parameter values

**PK parameters**	**Biological significance**	**Type Vicenza Mean value ± SD**	**Control Mean value ± SD**
*k*_rel_ (min^−1^)	(UL+HMW) multimers release constant	5.87^−2^ ± 3.49^−2^	2.74^−2^ ± 8.41^−3^
*k*_proteol_ (min^−1^)	(UL+HMW) to LMW multimers proteolysis constant	1.04^−3^ ± 2.54^−3^	4.59^−4^ ± 6.40^−4^
*k*_el_ (min^−1^)	(UL+HMW) and LMW multimers elimination constant	1.08^−2^ ± 5.22^−3^	1.17^−3^ ± 6.88^−4^
*Q* (U/kg)	Amount of VWF released	1.09^2^ ± 3.40^2^	4.47 ± 3.33
*T*_1/2_ (min)	VWF half‐life	64 ± 60	592 ± 846

*Note*: Including VWF release constant (*k*_rel_), proteolysis constant (*k*_proteol_), elimination constant (*k*_el_), amount of VWF released (*Q*), VWF half‐life (*T*_1/2_) and corresponding standard deviation (SD) as calculated according to Galvanin model in type Vicenza and healthy control subjects.

## 4 DISCUSSION

In this study, we examine whether it is appropriate to classify type Vicenza as a type 1 VWD, considering all of its known in vivo and in vitro features, also with the aid of a mathematical model. We conclude that it would be better to place type Vicenza VWD in a new type 2 class, emphasising the abnormalities peculiar of type Vicenza VWD, and introducing the interaction with clearance receptors as a new functional feature of VWF.

While the phenotype and genotype of type Vicenza VWD have been clearly defined [22], its classification has been subject of different proposals in the last decades, highlighting the difficulty of including type Vicenza in any of the known VWD groups. At present, it is included in type 1 VWD. According to the ISTH [1], type 1 VWD is a partial reduction of VWF without a significantly altered multimer pattern (especially without any loss of large VWF multimers), or any specific abnormalities in the behaviour of VWF towards ligand‐binding sites. This definition does not specify the contribution of VWF synthesis, although we generally assume that a quantitative VWF defect is a consequence of a defective synthesis. It has been suggested that an increase in FVIII:C ratio, as seen in our patients, is indicative of a decreased VWF synthesis [23]. However, several factors go against this conclusion. The normal platelet VWF content and the post‐DDAVP normalisation of circulating VWF levels, albeit with short‐lived effects [5], point to a normal synthesis and storage of type Vicenza VWF in the endothelial cells [16]. Confirmation would then come from the normal production of recombinant type Vicenza VWF, whether it is expressed at heterozygous or homozygous level, consistently with what Gézsi et al. described [13]. In contrast, Pruss et al. [24] report a mild decrease in recombinant p.R1205H VWF release in medium, but normal cell content levels, a picture suggesting an impaired VWF release that has never been demonstrated in type Vicenza patients.

The biochemical pathways influencing the concentration of VWF in the bloodstream, that is, its release, proteolysis and elimination, explored using a dynamic, physiology‐based mechanistic approach [21, 25] enabled us to demonstrate that it is not only the elimination rate that is significantly increased but also the rates of proteolysis and release of type Vicenza VWF [12, 25]. An increased proteolysis was unexpected because it is usually associated with a loss of large multimers (as seen in type 2A and type 2B VWD), whereas all VWF multimers are present in type Vicenza VWD, sometimes even the ultra‐large VWF forms. The reliability of this observation is confirmed by the VWF multimer composition seen both before and after DDAVP, which revealed a relative overabundance of LMW oligomers compared to the larger ones—in contrast with the picture seen in normal subjects, where the intermediate and large forms are predominant. Since HMW and LMW VWF multimers are eliminated at the same rate [26], and smaller VWF multimers are the result of proteolysis of larger ones, the multimer pattern identified confirms an increased VWF proteolysis in type Vicenza VWD. In an in vivo dynamic model, Gézsi et al. [13] explain the presence of ultra‐large VWF oligomers in type Vicenza VWD as the consequence of a very short VWF half‐life, short enough to interfere with the action of ADAMTS13 during the release of VWF from endothelial cells [13]. If this is the case, then in the steady state VWF elimination overrides its proteolysis in type Vicenza VWD, prompting the appearance of ultra‐large forms. In a static model, on the other hand, such as when recombinant VWF is expressed in vitro, no ultra‐large multimers are detectable. This goes to show that their presence does not depend on an abnormal multimeriation process or defective release of VWF in type Vicenza VWD, thus confirming Sadler's conclusions. It remains, however, to demonstrate whether the increased proteolysis and release of type Vicenza VWF are independent phenomena or are the consequence of its important increased elimination.

The increased elimination rate of type Vicenza VWF [21, 25] is most likely associated with an abnormal interaction between VWF and the binding sites involved in its clearance [26, 27]. In this setting, the p.R1205H mutation looks like a gain‐of‐function mutation that promotes VWF clearance by enhancing its interaction with its receptors [26]. The type Vicenza VWD picture is similar to that of type 2B in which VWF is synthesised and released normally, but its large VWF multimers disappear under the effect of proteolysis due to an increased interaction of VWF with platelet GPIb. It also resembles type 2A‐II VWD in which mutations in the A2 domain lead to an increased interaction between VWF and ADAMTS13, with consequent disappearance of large VWF multimers. The key question is whether VWF clearance [25, 27], mediated by a number of receptors on macrophages that have yet to be fully identified [28], can be considered one of the VWF functions—like its interaction with platelet GPIb, collagen, FVIII, and other receptors.

The appropriate classification of type Vicenza VWD is no mere taxonomic conundrum; it has major therapeutic implications. Indeed, patients carrying a quantitative VWF defect can be treated with DDAVP if they still have a residual synthesis of VWF with a normal or near‐normal structure, and a normal function. This is not the case in type Vicenza VWD patients, especially when a lasting haemostatic protection is required, because of their VWF's very short half‐life. Placing type Vicenza patients in the type 1C subgroup of VWD underscores the fact that they might not be good candidates for desmopressin, thus distinguishing them from other type 1 patients. But including type Vicenza in type 1C is a sort of a contradiction in terms: ‘type 1’ describes a residual synthesis of normal or functionally near‐normal VWF, while the extension ‘C’ suggests the presence of a functional defect—further emphasising the peculiarity of type Vicenza VWD.

In conclusion, the above considerations lead us to suggest that it would be better to classify as type 2 VWD all cases of type Vicenza and other type 1 forms involving a shorter VWF survival as the main contributor to the VWD phenotype. To do so, we need to decide whether or not the interaction between VWF and the receptors involved in its clearance is one of the functions of VWF.

## AUTHOR CONTRIBUTIONS

A. Casonato designed the research and wrote the paper. E. Galletta performed the haemostatic tests. F. Galvanin performed the mathematical modelling. V. Daidone performed the genetic analysis and analysed the data.

## CONFLICT OF INTEREST

The authors declare that there is no conflict of interest.

## Supporting information

Supporting Information

## Data Availability

The data that support the findings of this study are available from the corresponding author upon reasonable request.
