# Supplementary material for: Von Willebrand disease type Vicenza: In search of a classification for the archetype of reduced von Willebrand factor survival
Source: EJHaem. 2021 May 5;2(3):340–8. doi: 10.1002/jha2.196 (PMC9175995; doi:10.1002/jha2.196)
Supplement: Supplementary file 1 — Supporting Information [file JHA2-2-340-s001.docx]

**Supplementary Material**

**Pharmacokinetic model of Von Willebrand disease**

Physiologically-based compartmental models of VWD (Galvanin et al., 2014; Ferrari et al., 2018) are used to characterize in detail the mechanisms of release, proteolysis and clearance of VWF multimers that define the multimeric distribution of VWF in plasma. These models are characterized by a series of pharmacokinetic (PK) parameters that need to be estimated from individual VWF:Ag and VWF:CB data after DDAVP administration. In order to investigate further the mechanism of VWF elimination, proteolysis we propose the model illustrated in Figure 1.

**Figure 1** Structure of the reduced models used in this study. VWF:Ag and VWF:CB measurements are pointed out by coloured boxes (D =release of (UL+HMW) multimers after DDAVP administration; UL = ultra-large multimers; HMW = high molecular-weight multimers; LMW = low molecular-weight multimers).

The model includes two compartments, and aims to investigate the time evolution of VWF:Ag and VWF:CB concentrations in plasma. The models describes the time course of:

1. the combined release (D) of ultra-large (UL) and high molecular-weight (HMW) multimers;
2. the proteolysis of (UL+HMW) multimers to low molecular weight multimers (LMW) by means of ADAMTS13;
3. the multimer clearance, i.e. the elimination of multimers from plasma.

The underlying physiological assumptions are that at the basal state both (UL+HMW) multimers and LMW multimers are present, and that (UL+HMW) multimers can be cleaved to LMW multimers. It is assumed that the VWF:Ag measurements allow evaluating the total amount of VWF, i.e. the sum of (UL+HMW) and LMW multimers, whereas the VWF:CB measurements represent only high molecular weight (UL+HMW) multimers. Each subject is characterized using three PK constants, namely the multimer release rate *k_rel_* [min^-1^], the proteolysis rate *k_proteol_* [min^-1^], and the elimination rate *k_el_* [min^-1^]. In this model the elimination rate is assumed to be independent from multimer size, i.e. *k*_el_ is the same for both (UL+HMW) multimers and LMW multimers. The model is described by the following differential equations:

$\frac{dx^{UL+HMW}}{dt}=k_{0}D e^{-k_{rel}(t-t_{max})}-k_{proteol}\left( x^{UL+HMW}-x_{b}^{UL+HMW} \right)-k_{el}(x^{UL+HMW}-x_{b}^{UL+HMW})$ (1)

$\frac{dx^{LMW}}{dt}=k_{proteol}\left( x^{UL+HMW}-x_{b}^{UL+HMW} \right)-k_{el}\left( x^{LMW}-x_{b}^{LMW} \right)$ (2)

where $x^{UL+HMW}$ and $x^{LMW}$ are the numbers of (UL+HMW) and LMW multimer units [U] present in the plasma, subscript *b* refers to the basal state, *t* is the time [min], and *t_max_* is the time at which the release profile peaks [min]. In the model antigen concentration $y^{AG}$ [U/dL] and collagen binding concentration $y^{CB}$ [U/dL] are described as:

$y^{AG}=\frac{x^{UL+HMW}+x^{LMW}}{V_{d}}$ (5)

$y^{CB}=\frac{x^{UL+HMW}}{V_{d}}$ (6)

where *V_d_* = 40 mL/kg_bw_ is the approximated distribution volume following Menache at al. (1996). Basal conditions at *t* = 0 are defined by $x\left( 0 \right)=\left[ x_{b}^{UL+HMW} x_{b}^{LMW} \right]=\left[ y_{b}^{CB}V_{d} y_{b}^{AG}V_{d}-y_{b}^{CB}V_{d} \right]$. Following Galvanin et al. (2014), a correction was introduced in the definition of the collagen binding measurements in order to account for the different affinity of multimers to collagen observed in distinct VWD types:

$y^{CB'}=ky^{CB}\frac{y_{b}^{AG}}{y_{b}^{CB}}$ (7)

where *k* is a correction factor to be estimated from data. The overall set of parameters to be estimated from available DDAVP measurements is **θ** = $\left[ k_{rel} k_{proteol} k_{el} D ky_{b}^{CB} t_{max} \right]$. Parameter estimation is carried out using a maximum likelihood (ML) approach using the commercial software gPROMS^®^ (Process Systems Enterprise Ltd., London, UK).

The estimation of **θ** is done iteratively by solving a mathematical optimization problem, where a critical issue in parameter estimation is providing a reliable initial guess (**θ**_0_) to initialize the optimization. In this study, **θ**_0_ was fixed to the values reported in Ferrari et al. (2018) for average subjects (control and Vicenza). After the initialization step the parameter set for each model was estimated for the single subject based on the VWF:Ag and VWF:CB data that were measured for that subject assuming a standard deviation of measurement errors of σ_y_ = 2 U/dL for both VWF:Ag and VWF:CB. This value was corroborated by repeated measurements as illustrated in Galvanin et al. (2014). The “two-step” iterative parameter estimation procedure presented in Ferrari et al. (2018), based on single subject VWF:Ag and VWF:CB data, was applied. The procedure is carried out according to the following steps:

*Step 0* – all parameter sets are free to vary their initialization value;

*Step 1* – pharmacokinetic parameters ${[k}_{rel} k_{proteol} k_{el}]$ are estimated by fixing $\left[ D ky_{b}^{CB} t_{max} \right]$ at the value found in previous step;

*Step 2* – correction parameters $[ky_{b}^{CB}]$ and $t_{max}$ are estimated, fixing ${[k}_{rel} k_{proteol} k_{el} D]$ at the value found in previous step.

*Step 1* and *2* are repeated since the estimates do not vary significantly.

The parameter estimation results from ML are analysed in terms of estimated value and a posteriori statistics for each single subject, including likelihood value (objective function obtained from the optimisation), parameter standard deviations (SDs), confidence intervals and Student’s t-values. The model fitting performance is evaluated in terms of χ^2^ test (Bard, 1977).

The area under the curve (AUC) is used to compare the relative amount of UL + HMW and LMW multimers released [U] after DDAVP administration in control and Vicenza subjects. *AUC^UL+HMW^* and *AUC^LMW^* are numerically calculated in gPROMS from

${AUC}^{UL+HMW}=\int_{0}^{\tau} x^{UL+HMW}dt$ (8)

${AUC}^{LMW}=\int_{0}^{\tau} x^{LMW}dt$ (9)

where τ = 1440 min is the overall duration of the DDAVP test. The VWF half life T^1/2^ [min] and the total amount of VWF released [U/kg] are computed, respectively, from

$T^{1/2} {=\frac{log(2)}{k_{el}}e}^{-k_{rel}(t-t_{max})}$ (10)

$Q={\frac{1}{BW}\int_{0}^{\tau} k_{0}D e^{-k_{rel}(t-t_{max})}dt}$ (11)

where *BW* is the body weight [kg] of the subject.

Post-DDAVP average PK parameter values ${[k}_{rel} k_{proteol} k_{el}]$, amount of VWF released (*Q*), VWF half-life (*T*^1/2^) and corresponding SDs are calculated from the individual ML estimates for each group of subjects (Control/Vicenza).

**References**

1. Ferrari M, Galvanin F, Barolo M, Daidone V, Padrini R, Bezzo F, Casonato A. A mechanistic model to quantify von Willebrand factor release, survival and proteolysis in patients with von Willebrand disease. Thromb Haemost. 2018 Feb;118(2):309-319.
2. Galvanin F, Barolo M, Padrini R, Casonato A, Bezzo F. A model based approach to the automatic diagnosis of Von Willebrand disease. AIChE J 2014;60:1718–1727.
3. Menache D, Aronson DL, Darr F, Montgomery RR, Gill JC, Kessler CM, Lusher JM, Phatak PD, Shapiro AD, Thompson AR, White GC. Pharmacokinetics of von Willebrand factor and factor VIIIC in patients with severe von Willebrand disease (type 3 VWD): estimation of the rate of factor VIIIC synthesis. Br J Haematol 1996;94(04):740–745.
4. Process Systems Enterprise, gPROMS, www.psenterprise.com/gproms, 1997-2020.
5. Bard, Y. *Nonlinear parameter estimation*, Academic Press, Inc., New York, 1974.
